# A multi-species, multi-pathogen avian viral disease outbreak event: Investigating potential for virus transmission at the wild bird – poultry interface

**DOI:** 10.1080/22221751.2024.2348521

**Published:** 2024-04-30

**Authors:** Scott M. Reid, Alexander M. P. Byrne, Fabian Z. X. Lean, Craig S. Ross, Andrei Pascu, Richard Hepple, Maria Dominguez, Susanne Frost, Vivien J. Coward, Alejandro Núñez, Joe James, Levon Stephan, James N. Aegerter, Ian H. Brown, Ashley C. Banyard

**Affiliations:** a Virology Department, Animal and Plant Health Agency (APHA) Weybridge, Addlestone, UK; b Pathology and Animal Sciences Department, APHA Weybridge, Addlestone, UK; c APHA England Field Delivery, APHA Stafford, Stafford, UK; d APHA Bridgwater, Bridgwater, UK; e APHA England Field Delivery, Stroud, UK; f APHA Bakewell, Bakewell, UK; g WOAH/FAO International Reference Laboratory for Avian Influenza, APHA Weybridge, Addlestone, UK; h Veterinary Exotic Notifiable Disease Unit (VENDU), London, UK; i APHA Sand Hutton, National Wildlife Management Centre, York, UK

**Keywords:** High pathogenicity avian influenza virus (HPAIV); H5N8; clade 2.3.4.4b; multi-species, avian paramyxovirus type 1

## Abstract

A free-range organic broiler (*Gallus gallus domesticus*) premises in Staffordshire was infected by high pathogenicity avian influenza virus (HPAIV) H5N8 during the 2020–2021 epizootic in the United Kingdom (UK). Following initial confirmation of the infection in poultry, multiple wild bird species were seen scavenging on chicken carcasses. Detected dead wild birds were subsequently demonstrated to have been infected and succumbed to HPAIV H5N8. Initially, scavenging species, magpie (*Pica pica*) and raven (*Corvus corax*) were found dead on the premises but over the following days, buzzards (*Buteo buteo*) were also found dead within the local area with positive detection of HPAIV in submitted carcasses. The subacute nature of microscopic lesions within a buzzard was consistent with the timeframe of infection. Finally, a considerable number of free-living pheasants (*Phasianus colchicus*) were also found dead in the surrounding area, with carcasses having higher viral antigen loads compared to infected chickens. Limited virus dissemination was observed in the carcasses of the magpie, raven, and buzzard. Further, an avirulent avian paramyxovirus type 1 (APMV-1) was detected within poultry samples as well as in the viscera of a magpie infected with HPAIV. Immunohistochemistry did not reveal colocalization of avian paramyxovirus antigens with lesions, supporting an avirulent APMV-1 infection. Overall, this case highlights scenarios in which bi-directional transmission of avian viral diseases between commercial and wild bird species may occur. It also underlines the importance of bio separation and reduced access when infection pressure from HPAIV is high.

## Introduction

Clade 2.3.4.4b H5 high pathogenicity avian influenza virus (HPAIV) re-emerged across Europe during the winter of 2020-2021. Throughout this period, 300 H5Nx HPAIV detections were made in wild birds of various taxonomic orders in Great Britain (GB), dominated by subtype H5N8, often in wild waterfowl [1]. The clade 2.3.4.4b H5N8 HPAIV detected in Europe during this season demonstrated a close phylogenetic relationship to viruses detected in Russia, Kazakhstan, and Iraq earlier in 2020 [2]. Genetic variation of the different influenza segments of the H5Nx HPAIVs detected during this epizootic suggested there was exchange between high pathogenicity (HP) and low pathogenicity (LP) AIVs circulating in wild birds in Eurasia and Africa, resulting in the generation of multiple H5 subtypes and genotypes [1,2]. However, in the United Kingdom (UK), whilst multiple H5Nx subtypes were detected, H5N8 dominated with only a single H5N8 HPAIV genotype being defined [3]. Across GB, 24 cases of HPAIV were detected in poultry or captive bird premises during the 2020–2021 epizootic, of which 22 were due to H5N8 [4,5] and more than 2 million poultry were affected [1].

Prior to the emergence of H5Nx in GB, the risk of AIV being introduced into GB annually was associated with the arrival of overwintering migratory waterfowl carrying a variety of HP – and LPAIVs. Importantly, infection of wild waterfowl with HPAIV differs significantly from the disease outcomes seen in poultry. In wild bird species including widgeon and teal, infection and shedding of HPAIV in the absence of clinical disease may occur [6]. In contrast, infection of Galliformes (ground feeding land fowl) with the H5Nx clade 2.3.4.4b HPAIVs causes extensive mortality [7,8] and its emergence during the 2020–2021 season, and continued circulation since, has had a profound impact on all poultry sectors in the UK and Europe. Although the susceptibility of Galliforme species to HPAIV infection is well documented, limited information is available regarding the susceptibility of gamebirds including pheasants, and less still on most wild species, especially where these may have a role as bridging hosts; defined as a species competent for pathogen dissemination, having direct contact or a shared habitat with target populations [9]. Some populations of gamebirds that occur in abundance may provide a bridging role between poultry and the environment [10]. Whilst the exact pathways of incursion of HPAIV into poultry premises likely vary, faecal excretion of virus from wild waterfowl is proposed to be a key factor despite the absence of clinical disease in these species. Direct, or indirect viral transmission at the wild bird: poultry interface often results in transmission to poultry that cannot tolerate infection. Severe disease generally emerges with high mortality. Whilst transmission pathways are usually considered uni-directional, likely via infectious faecal matter disseminating virus from wild birds to poultry, bi-directional infection might also be possible with transmission of HPAIV from poultry to wild birds. The ongoing evolution and change in GS/GD HPAIVs with long-term maintenance in bird populations is influenced by virus introduction from domestic to wild birds especially where there is no bio separation in populations. As many LPAIVs circulate silently through avian populations, and some Anseriformes can be infected with HPAIV with mild clinical presentations [8], the movement of AIVs between poultry and wild birds likely occurs continuously. Disease is then only generally observed when HPAIV infection occurs in poultry although occasionally LPAIVs can cause clinical disease in poultry [11,12]. Further, where poultry are reared in open systems, freely sharing habitats with wild birds, the risk of such transmission increases.

Like AI, Newcastle disease (ND) is also a notifiable avian disease (NAD), affecting chickens and other avian species, caused by virulent strains of *Avian paramyxovirus* type 1 (APMV-1 [13,14]). Virulent APMV-1/ND virus was last reported in Great Britain (GB) in 2006 [13]. Unlike virulent strains of APMV-1, infection with avirulent strains of APMV-1 alone, which include transmitted vaccine viruses, generally cause subclinical infections or mild disease. However, APMV-1 infection can predispose or exacerbate disease with other concurrent respiratory pathogens.

Here, we detail an unusual instance of potential bi-directional transmission where wild birds have scavenged infected poultry carcasses. The disease event was followed by the detection of infection in wild bird populations including magpie, raven, buzzards and pheasants. We comment on the potential dynamic observed and the risk of different farmed poultry species as a source of HPAIV for infection of wild bird species. Interestingly, avirulent avian paramyxovirus type 1 (APMV-1) was also detected in one house.

## Materials and methods

### Epidemiologic description of the infected premises (IP)

**Figure 1.**
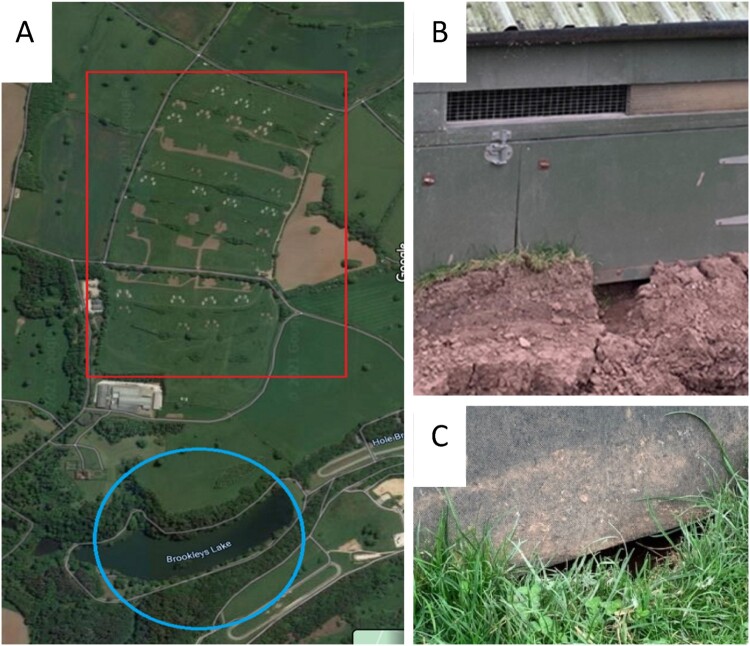
Ecological context of the site (A) (red rectangle denotes pasture, blue circle highlights the small lake) including evidence for wild animal and wild bird activity around poultry houses (B and C).

The IP was a multi-age, integrated broiler unit in the North of England [5] comprising five brooding houses and 40 free-range mobile rearing houses across three large pasture fields within a single estate of 4500 acres (Figure 1A). The premises housed approximately 49,000 birds, comprising 23,000 poults (less than four weeks old) and 26,000 older birds (over four weeks old). Poultry were finished on the farm and slaughtered at the estate’s own abattoir, approximately 1.6 km from the farm, accessible via private roads through the estate. Biosecurity surrounding the brooding houses was fair, with separate entrances for every brooding shed and disinfectant footbaths at shed entrances (Cyclex (http://disinfectants.defra.gov.uk/DisinfectantsExternal/Default.aspx?Module = ApprovalsList_SI) at a dilution of 1:24) with dedicated footwear being donned as necessary but without a change of personal protective clothing and an absence of visitor sign in and out registers. The biosecurity around the free-range area was poor with no disinfectant or dedicated clothing/footwear. Mobile houses were moved across the pasture range between batches (following depopulation) to provide a fresh area for the chickens.

The ecological context of the site was relatively simple, with its range (pasture; Figure 1A) sitting within a neighbourhood dominated by permanent grass and a small lake (<800 m from the range and mobile houses; Figure 1A). At a larger scale, the site sat at the southern edge of a range of low hills, with pasture as its main land use, along with some woodland which often covered the steeper valley sides. However, to the south and east, an extensive flat lowland landscape was dominated by arable landcover. Waterbodies, especially those large enough to support aggregations of wild waterfowl comprising >200 birds of mixed species, were largely limited to those associated with the hilly region, including a cluster of ponds, pools, and small lakes within the estate. Some ponds were as close as 350 m from the edge of the range although the most significant waterbody was the nearby lake, comprising approximately 6.5 hectares of open water and the largest within 3.3 km, which was set in grassy banks and was likely to be attractive to waterfowl; records suggest mean annual count of >500 susceptible waterbirds (waterfowl, gulls, some waders: [15]). Most of the other waterbodies within 3 km of the site were small, with banks lined by trees where they sat in steep-sided valleys and appeared relatively unattractive to waterfowl. Whilst waterfowl were likely to be locally abundant around the farm site, the generally small and unattractive character of waterbodies in the hilly landscape, and their relative paucity across the flat agricultural landscape suggested that waterfowl were not generally abundant. Otherwise, extensive woodland nearby may have hosted aggregations of corvids as well as abundant populations of passerines, and whilst the nearest known gull roost was >15 km distant, gulls were likely to be common across this working agricultural landscape. Of potential significance was the presence of an extensive theme park nearby (1.5 km), along with its own network of pools and ponds and opportunities for some species to exploit a rich source of anthropogenic forage, especially bridge species such as corvids, gulls and passerines. This was anticipated to sustain a more abundant population of bridge species than might have been expected from the natural and agricultural landcover available close to the farm site.

Potential intrusion of rodents or small wild bird appeared possible through ground-level holes worn in the soil beneath the mobile house (Figure 1B,C). Reports were made of wild birds, corvids, and pheasants, entering houses to exploit food, or attacking poultry through the ground level. Preceding and throughout the outbreak, poultry were permanently housed to comply with the Avian Influenza Housing Order (https://www.gov.uk/government/news/bird-flu-latest-situation-avian-influenza-prevention-zone-declared-across-great-britain) and the chickens had been confined to the houses since 14th December 2020.

The timing of this case may also have been significant, with bird behaviour being intermediate between the well understood overwintering state, producing a distinct use of the landscape, population structures and daily foraging behaviours, and the summer breeding state with different use of the landscape, population structures and foraging behaviours. In addition, the case occurred through one of the COVID-19 lockdown periods in the UK, with the theme park closed to visitors, and the consequential absence of anthropogenic forage.

A proportion of the wild bird carcasses were submitted from outside of the IP (exact locations not recorded) as estate workers such as gamekeepers brought dead pheasants from the wider estate to the IP entrance to hand over for submission to Animal and Plant Health Agency (APHA) Weybridge for testing. All samples were submitted as part of disease investigations at the IP.

### Sampling under suspicion of NAD

Suspicion of NAD was reported in chickens on the IP by a private veterinary surgeon on 26th March 2021 and official sampling was undertaken by an APHA veterinarian later that day for overnight submission to the National Reference Laboratory for AIV. For the initial statutory investigation, matched cloacal swabs (C: 20 per shed), oropharyngeal swabs (OP: 20 per shed), two fresh carcasses with OP and C swabs collected from each, and clotted blood samples (20 per shed) were submitted from three houses: House 10 (22/22/20); House 15 (22/22/20) and House 9 (20/20/20). No carcasses were submitted from House 9 from the initial statutory investigation.

Two additional chickens from House 9, along with one dangerous contact raven (*Corvus corax*) and two pheasants (*Phasianus colchicus*) were further received for NAD diagnostic investigation on 29th March. Six non-poultry carcasses from four pheasants, one buzzard (*Buteo buteo*), and one magpie (*Pica pica*) found in the immediate vicinity outside of the affected poultry houses and within the poultry range areas where free ranging would have occurred if permitted, were also collected, and sampled on 29th March. Blood samples were not available for serological testing of the non-poultry species.

### Pathological assessment

Poultry carcasses and submitted wild bird carcasses were examined in accordance with NAD in GB [4]. Gross pathological assessment was undertaken on all submitted carcasses. Where necessary, tissues were fixed in 10% (v/v) buffered formalin for both haematoxylin and eosin (H&E) staining as well as influenza A virus-specific and avian paramyxovirus immunohistochemical (IHC) staining using an anti-nucleoprotein (NP) monoclonal antibody [7,16]. A semi-quantitative scoring system ranging from 0 (absence of staining) through to 4 (abundant staining) was applied for IHC and similarly from 0 (absence of lesions) through to 4 (marked changes) for H&E staining. The details of the classification criteria are as described by Lean et al. [17].

### Virological investigation

All samples from the poultry and non-poultry species were processed for RNA extraction [18], and RNA was then tested for the presence of AIV nucleic acid using a suite of four real-time reverse transcription polymerase chain reaction (rRT-PCR) assays including a Matrix (M)-gene assay for generic influenza A virus detection [19]; specific detection of H5 – [20] and H7-AIVs [21]; and an N8-specific rRT-PCR to confirm the AIV neuraminidase type [22]. In each assay, a positive result was denoted by a Cq value ≤36.0. The samples were also screened for APMV-1 by an rRT–PCR assay using primers and probes targeting the large polymerase (L) gene [23]. APMV-1 testing was undertaken in parallel with the AIV assays as is routine for statutory NAD investigations in GB. Although non-poultry species were not routinely tested for APMV-1, the decision was made to test these species from this case along with the poultry species. A positive result with the APMV-1 rRT-PCR assay was denoted by a Cq value ≤37.0. All amplifications were carried out in an AriaMx or MxPro qPCR System (Agilent).

Virus isolation was attempted in 9 – to 11-day-old specific pathogen-free (SPF) embryonated fowls’ eggs on all four standard pooled tissues (brain, intestinal contents, lung and trachea, and mixed viscera) collected from submitted carcasses and on pools of five oropharyngeal or cloacal swabs according to the internationally recognized methods [24].

### Sequencing and phylogenetic analysis

Conventional RT–PCR and Sanger sequencing were carried out for the cleavage site (CS) on the haemagglutinin (HA) gene of selected H5 AIV PCR-positive samples as described previously [20,24]. Sanger sequencing was also undertaken using APMV-1 fusion (F)-gene-specific primers to produce a 374 bp product as previously described [25] on selected APMV-1 rRT-PCR-positive samples from House 9. For whole genome sequencing (WGS), viral RNA was processed and sequenced to generate a consensus sequence as described previously [3]. Contemporary H5N8 HPAIV sequences were obtained from the GISAID EpiFlu Database (https://www.gisaid.org/), whilst ND F-gene sequences were obtained from the NDV consortium sequence database (https://github.com/NDVconsortium/NDV_Sequence_Datasets). Sequences were aligned and used to infer Maximum-likelihood phylogenetic trees as described previously [13]. The three full-genome H5N8 sequences generated as part of this study are available through the GISAID EpiFlu Database, under the following accession numbers: EPI_ISL_11406397, EPI_ISL_11406400 and EPI_ISL_11406449. The NDV F-gene sequence are available through Genbank, under the following accession number: OQ674031.

### Routine serology for AIV subtypes H5 and H7, and APMV-1

Sera collected from clotted bloods were screened by haemagglutination inhibition tests (HAIT) to detect virus subtype-specific antibodies against H5 and H7 AIV antigens, and APMV-1 as described previously [26,27]. Serum samples with a reciprocal HI titre greater than or equal to 16 were considered positive [26,27].

## Results

### Disease emergence in broiler poultry and wild birds

Suspicion of NAD was initially reported on 21st March 2021 when clinical signs were observed across eight sheds with 10 deaths recorded. Twenty further deaths were noted on 22nd March and by the afternoon of 23rd March, 100 further deaths had been recorded. The clinical disease presentation included greenish diarrhoea, purple combs, and ruffled feathers, with neck and head tremors, torticollis and wing flapping. On 26th March, clinical signs and increased mortality had also been observed across three houses (3, 4, and 6) and three houses (5, 7, and 8) remained unaffected at the time of culling. Elevated mortality (approximately 50%), lethargy, cyanotic combs, reddened legs, neurological signs followed with birds succumbing to disease. Six carcasses were submitted initially to APHA-Lasswade for post-mortem examination (PME) where no gross pathological lesions were observed. The official samples were received at APHA Weybridge on 27th March for statutory testing for NAD as well as further pathological assessment.

Following report case confirmation, wild birds were noted on and around the premises with some interacting with chickens exhibiting clinical disease. A raven was found dead one day before official samples were submitted (26th March) and a few days after the confirmation of disease on the site, numerous pheasants released local to the site were found dead. Pheasant mortality increased significantly in the days following the first detection of a positive pheasant for HPAIV H5N8.

### Virological investigation in House 10, House 15, and House 9, and wild birds

All chickens sampled from House 10 (*n* = 22/22) and House 15 (*n* = 22/22) were positive for influenza A viral RNA by M-gene screening rRT-PCR, and positive by both H5- and N8-specific AIV rRT-PCR assays. Each swab was rRT-PCR-negative for subtype H7 AIV and APMV-1, and corresponding birds were seronegative for antibodies towards AIV subtypes H5 and H7 as well as to APMV-1 (data not shown).

In House 9, only a single bird (*n* = 1/20) was rRT-PCR-positive for AIV H5N8, but 17/20 birds were rRT-PCR-positive for APMV-1, and 11/20 birds were seropositive to APMV-1 (Table 1). These chickens were broilers and would therefore not normally have been vaccinated against APMV-1.

**Table 1.** Real-time RT-PCR and ND serology results from the chickens in House 9. All swabs were negative for influenza A (M-gene) and H7-specific rRT-PCR assays. A single bird was positive by the H5- and N8-specific rRT-PCRs. Positive results are denoted in bold.

Bird number	APMV-1		H5		N8		ND Serology
	C^a^	OP^b^	C	OP	C	OP	
1	NEG^c^	**POS^d^**	NEG	NEG	NEG	NEG	**16^e^**
2	**POS**	**POS**	NEG	NEG	NEG	NEG	**16**
3	**POS**	**POS**	NEG	NEG	NEG	NEG	8
4	**POS**	**POS**	NEG	NEG	NEG	NEG	IS^f^
5	**POS**	**POS**	**POS**	NEG	**POS**	NEG	4
6	**POS**	**POS**	NEG	NEG	NEG	NEG	**64**
7	**POS**	**POS**	NEG	NEG	NEG	NEG	<2
8	NEG	NEG	NEG	NEG	NEG	NEG	**64**
9	**POS**	**POS**	NEG	NEG	NEG	NEG	**64**
10	**POS**	**POS**	NEG	NEG	NEG	NEG	**64**
11	**POS**	NEG	NEG	NEG	NEG	NEG	8
12	**POS**	**POS**	NEG	NEG	NEG	NEG	**64**
13	**POS**	NEG	NEG	NEG	NEG	NEG	**64**
14	NEG	NEG	NEG	NEG	NEG	NEG	**64**
15	**POS**	**POS**	NEG	NEG	NEG	NEG	**64**
16	NEG	NEG	NEG	NEG	NEG	NEG	**32**
17	**POS**	**POS**	NEG	NEG	NEG	NEG	2
18	**POS**	**POS**	NEG	NEG	NEG	NEG	2
19	**POS**	**POS**	NEG	NEG	NEG	NEG	8
20	NEG	**POS**	NEG	NEG	NEG	NEG	8

^a^ C, cloacal swab.

^b^ OP, oropharyngeal swab.

^c^ NEG, negative result.

^d^ POS, positive result.

^e^ Reciprocal HI titres of 16 or higher were positive.

^f^ IS, insufficient volume of serum available for testing.

As well as the chickens from House 10, House 15, and House 9 sampled by the APHA veterinarian on 26^th^ March (Table 1), rRT-PCR molecular assessment of pooled brain tissue taken at PME from the additional chickens from House 9 also demonstrated positivity for both AIV subtypes H5 and N8 (Table 2). Positivity for AIV subtypes H5 and N8 was also shown across the additional samples taken from the two pheasants, raven, buzzard, and magpie (Table 2). Interestingly, the magpie was also positive for APMV-1 in the viscera tissues. All other samples collected at PME were rRT-PCR-negative for APMV-1 (Table 2).

**Table 2.** Summary of the rRT-PCR results for the samples taken from poultry and non-poultry species at PME including the official samples collected from the chickens in House 10 and House 15 from the initial statutory investigation, and the additional carcass samples collected from the chickens (House 9), raven, pheasants, buzzard and magpie.

rRT-PCR for specific detection of H5 – and N8-specific AI viral RNA																
Species	Chicken (House 10)		Chicken (House 15)		Chicken (House 9)		Raven		Pheasant		Pheasant		Buzzard		Magpie	
Sampling Date	27/03/2021				29/03/2021		26/03/2021		29/03/2021		01/04/2021		01/04/2021		01/04/2021	
Number of Carcasses	2		2		2		1		2		4		1		1	
PCR assay ^c^	**H5**	**N8**	**H5**	**N8**	**H5**	**N8**	**H5**	**N8**	**H5**	**N8**	**H5**	**N8**	**H5**	**N8**	**H5**	**N8**
Brain ^c^	POS^a^	POS	POS	POS	POS	POS	POS	POS	POS	POS	POS	POS	NEG^b^	NEG	POS	POS
Intestine ^c, d^	POS	POS	POS	POS	NEG	NEG	POS	POS	POS	POS	POS	POS	NEG	NEG	POS	POS
Lung and trachea ^c^	POS	POS	POS	POS	NEG	NEG	POS	POS	POS	POS	NEG	NEG	POS	POS	POS	POS
Visceral ^c, e^	POS	POS	POS	POS	NEG	NEG	POS	POS	POS	POS	NEG	NEG	POS	POS	NEG	NEG
Oropharyngeal swab (bird 1)	POS	POS	POS	POS	NEG	NEG	ND^f^	ND	POS	POS	ND	ND	ND	ND	ND	ND
Oropharyngeal swab (bird 2)	POS	POS	POS	POS	NEG	NEG			NEG	NEG						
Cloacal swab (bird 1)	POS	POS	POS	POS	NEG	NEG	POS	POS	POS	POS	ND	ND	ND	ND	ND	ND
Cloacal swab (bird 2)	POS	POS	POS	POS	NEG	NEG			NEG	NEG						
**rRT-PCR for generic detection of APMV-1 RNA**																
Swabs and tissues	NEG		NEG		NEG		NEG		NEG		NEG		NEG		POS (viscera)	

^a^ POS, positive result.

^b^ NEG, negative result.

^c^ Tissues were pooled if two or more of the same species or same shed were sampled.

^d^ Intestine represents pooling of jejunum and caecal tonsil if present for the species.

^e^ Visceral tissues represents pooling of heart, liver, spleen, and kidney.

^f^ ND = not determined.

### Confirmation of HPAIV in chickens in House 10 and House 15

To confirm HPAIV H5 infection, CS sequencing was undertaken on a selection of samples from House 10 (*n* = 3) and House 15 (*n* = 3) and was demonstrated to contain multiple basic amino acids. All sequences from the samples were identical over the 250 nucleotides with the CS sequence motif (PLREKRRKRGLF) being identical to the vast majority of HPAIVs detected in the UK and across Europe during the 2021 season.

**Figure 2.**
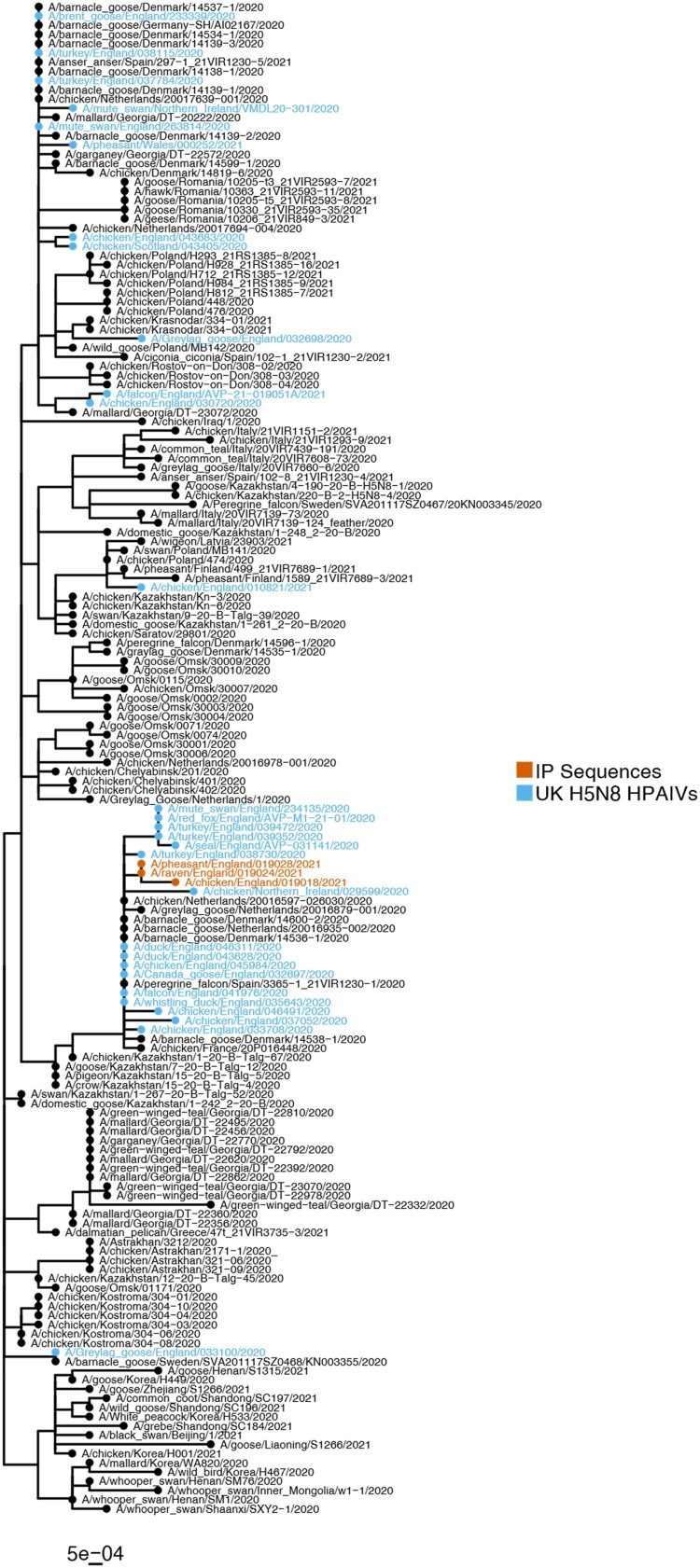
AIV H5N8 sequences obtained from the IP demonstrated similarity to UK and global H5N8 sequences from 2020 to 2021. Maximum-likelihood phylogenetic tree of the HA gene from global H5N8 sequences from 2020 to 21. Sequences obtained from the IP and the UK are highlighted by coloured tip shapes.

WGS of an isolate obtained from pooled tissues/swabs from chickens in House 10, along with isolates obtained from a pheasant carcass and the raven carcass demonstrated that the HA gene from these samples was highly similar forming a separate phylogenetic cluster (Figure 2). These sequences not only displayed high similarity to each other (>99.88% nucleotide identity), but also with other UK H5N8 HPAIV sequences from the 2020/21 epizootic (>99.06% nucleotide identity), which was also observed across the remaining influenza genes (>99.82% and >98.66% nucleotide identity to each other and UK H5N8 sequencies from 2020 to 2021, respectively).

### Confirmation of APMV-1 in House 9

**Figure 3.**
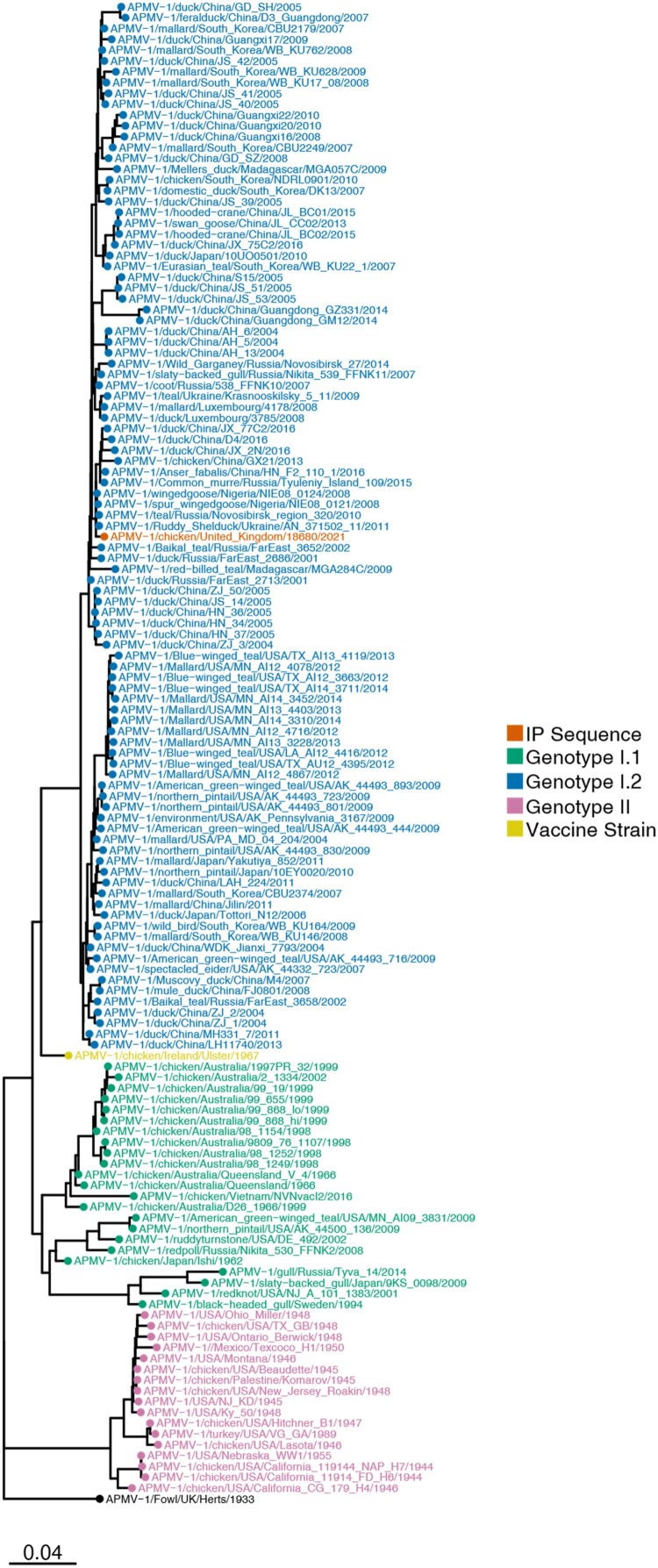
APMV-1 sequences obtained from the IP demonstrated it to be a genotype I.2 isolate clustering with global genotype I.2 sequences. Maximum-likelihood phylogenetic tree of the F-gene from global APMV-1 sequences. Sequences from different APMV-1 genotypes and that obtained from the IP are highlighted by coloured tip shapes.

APMV-1 F-gene sequencing data detected the presence of an avirulent CS within the F protein (G^113^KQGR/L^117^). Analysis of the F-gene demonstrated that this was a class II genotype I.2 (previously genotype 1b) APMV-1 (Figure 3), commonly associated with wild waterfowl [28].

### Post-mortem, histopathological, and viral immunohistochemical analyses

**Figure 4.**
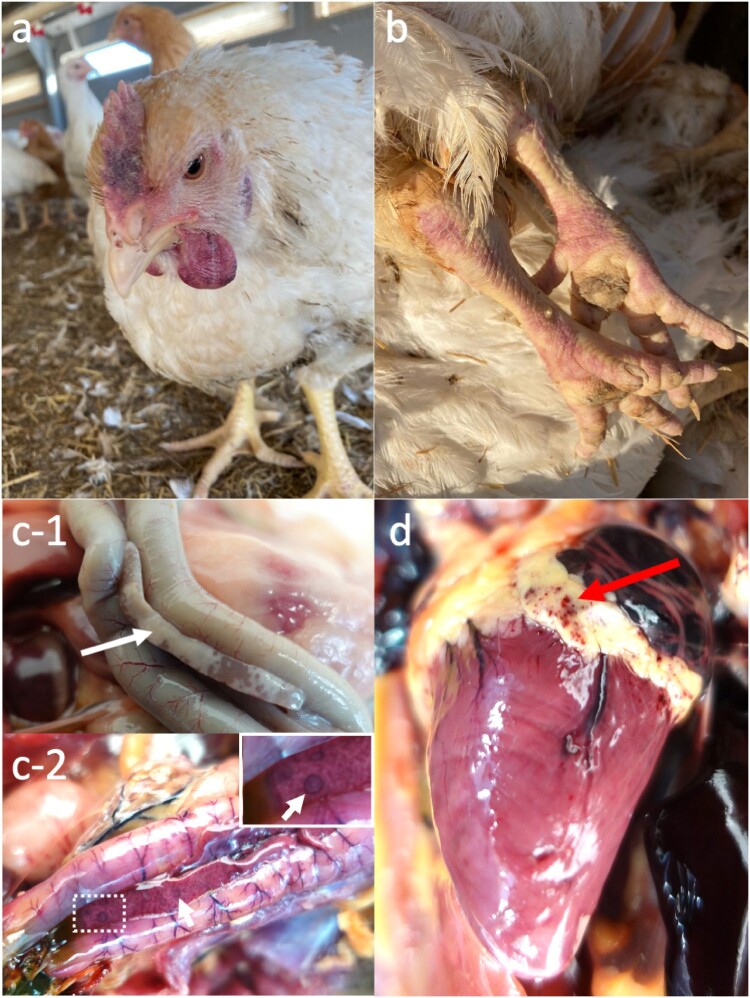
Gross pathological lesions detected within the HPAIV outbreak in a free-range organic broiler farm and associated wild bird infection. On-farm inspections of revealed cyanotic comb (a) and redenned legs (b). PME identified pancreatic necrosis (c; white arrows) in the broilers (c-1) and pheasants (c-2), and epicardial petechiae in the pheasant (d; red arrow).

Two birds from each House 9, 10, and 15, were submitted for PME. All carcasses were in good body condition. Chickens from House 10 and House 15 showed consistent splenomegaly (*n* = 4/6) which were not specific for HPAIV. Chickens from House 9 exhibited macroscopic changes including cyanosis around the face and feet (*n* = 2/2; Figure 4a,b, similar to that observed at ante-mortem), pancreatic necrosis (*n* = 2/2; Figure 4c-1), splenic necrosis (*n* = 1/2), and muscle petechiae (*n* = 2/2). As for the wild birds examined, gross pathological changes were noted from the pheasants which included pancreatic necrosis (1/6; Figure 4c-2), hydropericardium (1/6), pneumonia (3/6), epicardial petechiae (2/6; Figure 4d). The buzzard had mild hydropericaridum. Both the magpie and raven were unremarkable on necropsy (Supplementary Figure 1).

**Figure 5.**
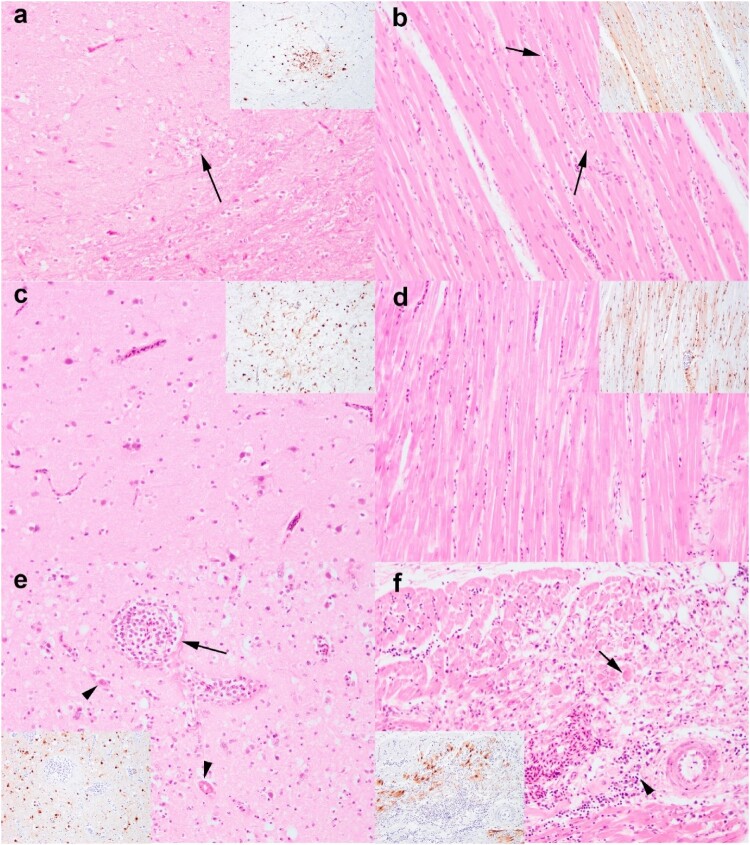
Comparative neuro- and cardiomyopathology of chickens (a, b), pheasants (c, d) and buzzard (e, f) infected with HPAIV H5N8. Mild neuronal necrosis with neuropil rarefaction (a) and co-localization of virus antigens (inset). Histological unremarkable brain from pheasant (c). Both chicken and pheasant have neuro and vascular tropism (c, d). Buzzard has lympho-plasmacytic perivascular cuffing (arrow) and oedema, endothelial hypertrophy and necrosis, thrombi deposition (black arrow heads), dispersed degenerate neutrophils in neuropil and neuronal necrosis (e) with viral neurotropism (inset). Mild myocardial degeneration in chickens (b) and absent in pheasant (d). Marked myocardial degeneration and disorganized myofibres along with lymphocytic infiltration in the buzzard (f). Images were taken at 200x magnification. H&E and IHC are from serial sections. Images were taken at 200x magnification.

Immunohistochemistry against AIV nucleoprotein revealed epithelial tropism in the respiratory and gastrointestinal tract, as well as neuro (Figure 5), myo, vascular, and lymphoid tropism of HPAIV in the chickens and pheasants (Supplementary Figure 2), confirmed a multisystemic infection consistent with HPAIV infection. The most consistent lesion across both chickens and pheasants are the severe pancreatic necrosis with intralesional virus antigens, with the lesions being acute. Generally, virus antigens were more abundant among the pheasants than the chickens particularly in the brain (Figure 5a,c) and spleen.

In contrast, while there was limited presence of virus antigens in the buzzard, magpie, and raven, apart for the moderate to abundant in the brain. Pancreatic and brain lesions were minimal to mild in the magpie and raven. The buzzard, however, presented with subacute myocarditis and encephalitis (Figure 5e,f). In addition to HPAIV detection by molecular virology, APMV-1 was detected in the magpie. Additional IHC was conducted on the magpie against avian paramyxovirus nucleoprotein which did not reveal presence of virus antigens, and therefore the encephalitis was only attributed to HPAIV infection.

## Discussion

Globally, outbreaks of HPAIV have significantly affected wild birds and poultry producers during both 2020–2021 and 2021/−2022 epizootic seasons. Where incursion into farmed poultry or backyard flocks is concerned, the dynamics of infection are generally considered to involve introduction of infectious material from a wild bird reservoir with the faecal-oral route of infection thought to be the most likely mechanism of transmission. Infection of wild birds with HPAIV can have a range of clinical disease outcomes and it is understood that several species of wild waterfowl are able to tolerate infection with HPAIV and can excrete, and therefore spread, infectious HPAIV in the absence of clinical disease. This study details a NAD outbreak event of HPAIV infection in a free-range poultry setting with evidence of wild bird activity that may have led to bi-directional infection.

Initial suspicion and subsequent detection of HPAIV in the housed, free-range broiler poultry premises during the 2020–2021 HPAI epizootic had similarities to other infection events within poultry. The clinical presentation was typified with neurological signs and a rapid rise in mortality within infected houses. At the time of the statutory investigation, all birds were housed in accordance with the Housing Order enforced on 14th December 2020 as infection pressure within the environment through wild bird infection was high. The most likely route of infection of the broilers was either through direct or indirect contact with wild birds as there were no concurrent HPAIV H5N8-infected IPs confirmed or under investigation in the UK. Also, no anthropogenic links to other affected premises could be established as part of the investigation, such as common food or bedding supply, linked maintenance activity, or shared employees. Holes around the base of the houses were reportedly created by wild birds (corvids, pheasants) digging in from outside in search of food and chickens inside scratching the ground. Rodent activity could also have been involved. The opening on the side of the houses to provide natural light and ventilation were covered by mesh, and sufficient to prevent access of small birds such as passerines. Gaps in some of the poultry housing and the sub-optimal biosecurity meant that wild birds, including free-living pheasants and scavenging bridge species (gulls and corvids), were able to access food within the houses. As a result of this scenario, following the incursion of HPAIV into the broilers, a direct mechanism of virus transmission had potentially emerged to wild and semi-feral birds present outside of the housed poultry. Furthermore, once infection had taken place within the chicken population on the premises, scavenging or predatory wild birds were seen attacking sick chickens, thereby potentially providing an unusual transmission route from housed poultry to free-living wild birds.

HPAIV H5N8 was detected in all sampled chickens in House 10 and House 15 but in only one chicken from House 9 that lacked serological reactivity to AIV antigens. However, pathological analysis of two chickens from House 9 revealed gross lesions, including pancreatic necrosis, along with the detection of intra-lesional viral antigens, consistent with HPAIV infection. The discrepancies between molecular and pathological analysis are likely due to sampling errors that have affected viral RNA integrity and subsequent detection. Nucleotide sequencing revealed the strong level of genetic identity of virus sequences from House 10, House 15 and the sequences obtained from the different wild bird species (Figure 2). After the initial incursion of HPAIV H5N8 into Europe in late 2020, the virus underwent rapid genetic diversification, including reassortment with LPAIVs present in the wild bird population, resulting in the generation of multiple H5 subtypes, and genotypes therein [1,2]. In terms of HPAIV H5N8, whilst multiple distinct genotypes were observed in Europe [1], in GB, all H5N8 sequences generated from wild birds and poultry represented a single genotype with high genetic similarity [3]. However, even in the context of this high degree of genetic relatedness, the three virus sequences obtained from poultry and wild birds on the IP investigated in this report clustered together across all gene segments discreetly from other GB viral sequences. This suggests the viruses obtained from the IP were distinguishable genetically from viruses reported elsewhere and supports the possibility of bi-directional transmission between wild birds and poultry.

Naturally infected pheasants were previously thought to be uncommon, and the pathology associated with 2.3.4.4 H5Nx natural infection has not been described [32]. In our study, the gross and histological lesions in the pheasant were comparable to the chickens. However, the HPAIV antigen loads were much more abundant in those from the pheasants than those from the chickens. This species-specific discrepancy has been noted in previous reports following experimental inoculation with H5N8 and other 2.3.4.4 H5Nx [29], and may be linked to increased viral replication in pheasants but warrants further investigation.

Birds of prey or scavenging birds generally exhibit limited systemic dissemination of HPAIV as opposed to poultry [30,31]. In this study, the course of infection in the corvids was peracute in the absence of apparent histological changes but virus antigens were detected in the brain, which could be implicated in functional changes in the brain. However, the buzzard exhibited peracute viral encephalitis and myocarditis, suggestive of a more protracted course of disease to allow lesions to develop [30]. Nevertheless, in addition to buzzards, corvid species could also act as useful sentinel species.

The detection of avirulent APMV-1 in House 9 was an unusual observation. Sequencing of the F-gene demonstrated that this was a class II genotype I.2 virus usually found in wild waterfowl. The birds that were positive for APMV-1 were unaffected by HPAIV except for a single bird, which was weakly positive for H5N8 (Table 1). Significantly, the clinical picture within this house was different to that seen in the other houses. Instead of the severe neurological disease, birds were noted as being subdued with a greenish or whitish diarrhoea. From a serological perspective, 11 birds were seropositive with moderate titres against APMV-1, consistent with a relatively recent infection with APMV-1. Interestingly, three birds were negative for APMV-1 RNA by rRT-PCR, but were seropositive, suggesting these birds were convalescing. Currently, within GB, the only licenced vaccine containing a genotype I.2 APMV-1 strain uses the strain Ulster 2C. Comparison of Ulster 2C F-gene with the APMV-1 F-gene propagated here demonstrated a 96% similarity (1594/1662 nucleotide identity), with the propagated isolate much more closely related to wild bird isolates observed in spur-winged goose Nigeria (99.5% identity (HG326605-8)), Mediterranean gull/Ukraine (99.4% identity (MZ101340)), teal/Novosibirsk (99.3% identity (KX352836)) and tadorna/Ukraine (99.3% identity (MZ101338)), all much more closely related to the strain identified in this study than the vaccine strains. These avirulent genotype I.2 APMV-1s are common in wild birds [28,32], and from this analysis, it appears likely that this isolate was most likely introduced through interactions with wild birds, not through spread of a vaccine strain. However, as Corvids are rarely described as harbouring these viruses, these species may have acquired their infection on this IP from poultry.

Fundamentally, the increase in global poultry production, particularly free-range systems, comes with heightened risk of AI incursion, and therefore biosecurity levels must improve accordingly [33,34]. Minor gallinaceous species including members of the Phasianidae, and Passeriformes have been suggested to act as bridge species between wildlife and poultry populations, given the current free-range nature of farming combined with recreational purposes and wild roaming habitats [35–37]. Experimental infection of ring-necked pheasants demonstrated that this species could also sustain virus shedding with transmission to contact-exposed birds, which may enable unrecognized dissemination and adaptation [36]. Nevertheless, improvements in biosecurity such as limiting wild bird access of potential bridge species to poultry houses, are necessary to reduce the risk of virus incursion, particularly in free-range farms [38].

## Supplementary Material

Supplementary_figures
