# Supplementary material for: A multi-species, multi-pathogen avian viral disease outbreak event: Investigating potential for virus transmission at the wild bird – poultry interface
Source: Emerg Microbes Infect. 2024 Apr 30;13(1):2348521. doi: 10.1080/22221751.2024.2348521 (PMC11168234; doi:10.1080/22221751.2024.2348521)
Supplement: Supplementary_figures [file TEMI_A_2348521_SM6984.docx]

**Supplementary information**

**
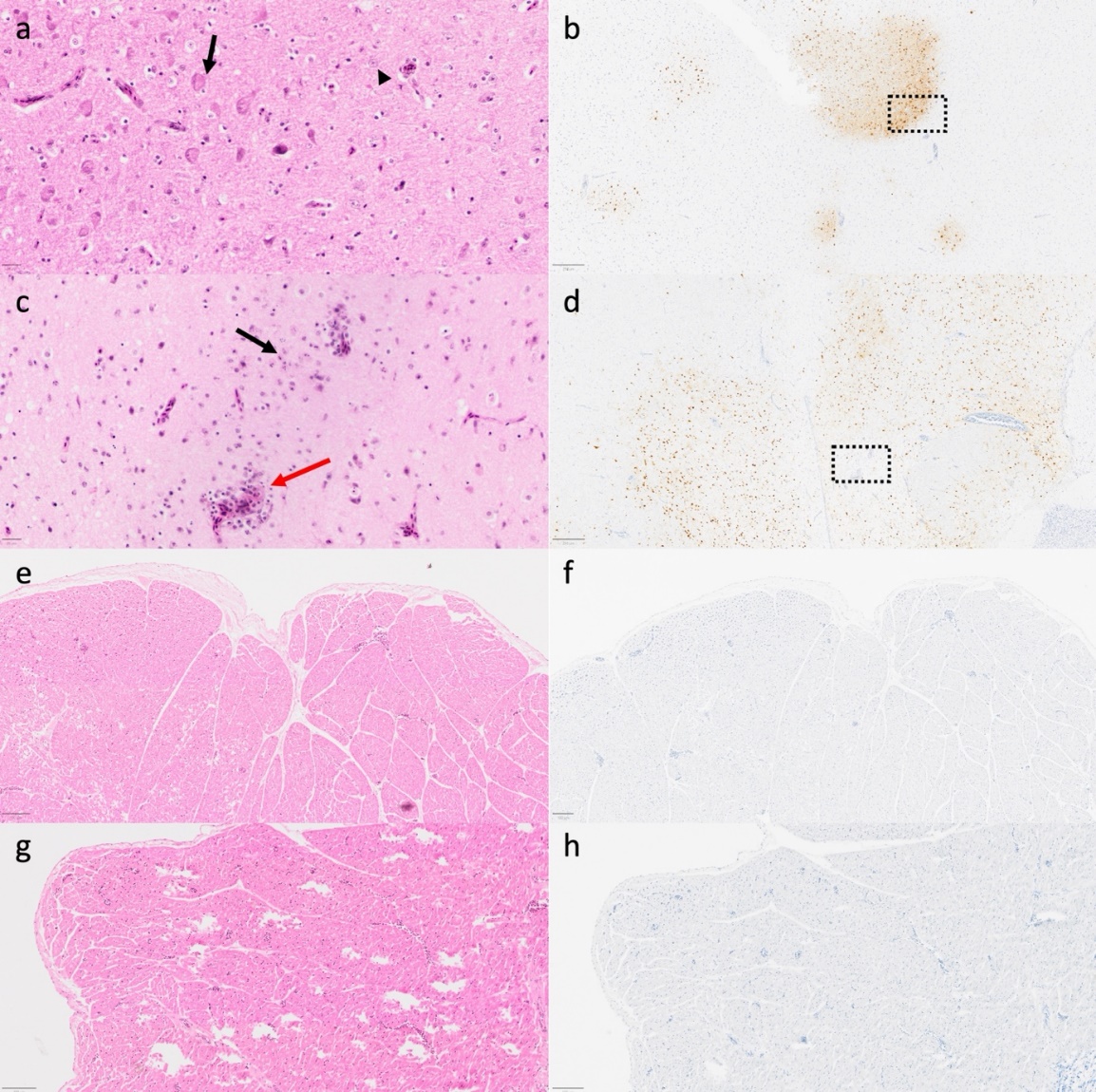
**

**Supplementary Figure 1. Comparative histopathology of raven (a, b, e, f) and magpie (c, d, g, h) infected with HPAIV H5N8.** In both the raven and magpie, there were rare areas of neuronal degeneration characterised by neuronal swelling (a; black arrow), neuronal necrosis (c; black arrow), or lymphocytic vasculitis (c; red arrow). This is in contrast to the multifocal to confluent immunolabeling of the neuronal parenchyma (b, d; areas marked by black dashed line represent areas of viral immunolabeling colocalised to histological lesion in a and c). In other organs, including the heart, there was no histological evidence of inflammation or degeneration (e, g), and virus antigen was not detected (f, h). H&E and IHC are from serial sections. The clear spaces within the myocardium (g) represent histological artifacts.


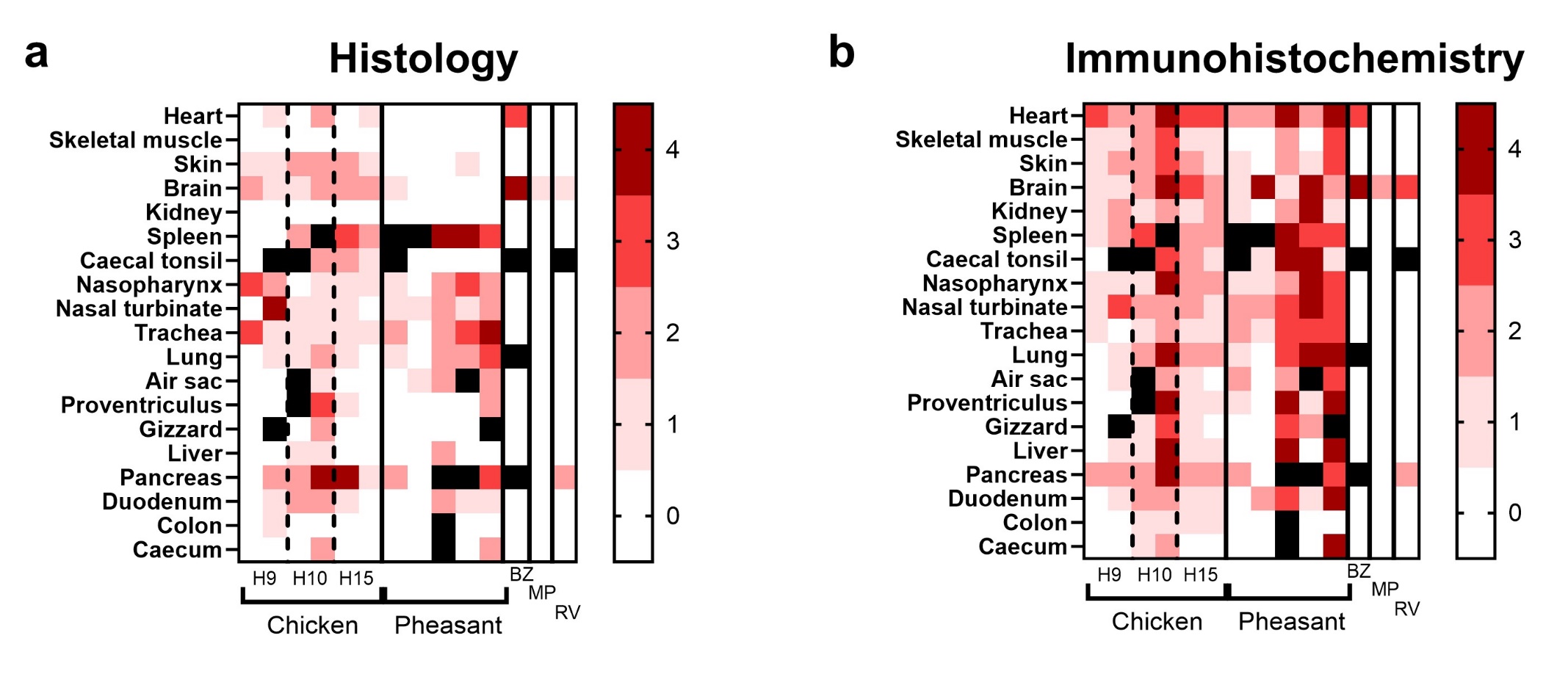


**Supplementary Figure 2.** **Histology and immunohistochemistry findings in chicken (Gallus gallus domesticus), pheasant (Phasianus colchicus), buzzard (Buteo buteo), magpie (Pica pica) and raven (Corvus corax).** Categorical heat maps indicated the maximum severity of histopathological changes (a) and the level of immunohistochemical labelling against Influenza A nucleoprotein (b). For histopathology: 0 absent; 1 minimal; 2 mild; 3 moderate; 4 marked; for immunohistochemistry: 0 absent; 1 rare; 2 scattered; 3 confluent; 4 abundant. Black indicates where no samples were available for analysis. Abbreviations: House 9 (H9), House 10 (H10), House 15 (H15), buzzard (BZ), magpie (MP), raven (RV).
